# Role of Gastric Microorganisms Other than *Helicobacter pylori* in the Development and Treatment of Gastric Diseases

**DOI:** 10.1155/2022/6263423

**Published:** 2022-03-14

**Authors:** Xiaoyan Duan, Ping Chen, Xiaoxia Xu, Meiling Han, Jianbo Li

**Affiliations:** ^1^Department of Gastroenterology, Affiliated Hospital of Inner Mongolia Medical University, Hohhot 010050, China; ^2^Inner Mongolia Key Laboratory of Molecular Imaging, Hohhot, 010050, China

## Abstract

The microenvironment in the stomach is different from other digestive tracts, mainly because of the secretion of gastric acid and digestive enzymes, bile reflux, special mucus barrier, gastric peristalsis, and so on, which all contribute to the formation of antibacterial environment. Microecological disorders can lead to gastric immune disorders or lead to the decrease of dominant bacteria and the increase of the abundance and virulence of pathogenic microorganisms and then promote the occurrence of diseases. The body performs its immune function through innate and adaptive immunity and maintains microbial balance through the mechanism of immune homeostasis. Microecological imbalance can lead to the invasion of pathogenic microorganisms and damage mucosal barrier and immune system. The coexistence of gastric microorganisms (including viruses and fungi) may play a synergistic or antagonistic role in the pathogenesis of gastric diseases. Probiotics have the ability to compete with intestinal pathogens, increase the secretion of immunoglobulin A (IgA), stimulate the production of mucin, bacteriocin, and lactic acid, regulate the expression and secretion of cytokines, and regulate the growth of microbiota, which all have beneficial effects on the host microbial environment. At present, most studies focused on *Helicobacter pylori*, ignoring other stomach microbes and the overall stomach microecology. So, in this article, we reviewed advances in human gastric microecology, the relationship between gastric microecology and immunity or gastric diseases, and the treatment of probiotics in gastric diseases, in order to explore new area for further study of gastric microorganisms and treatment of gastric diseases.

## 1. Introduction

**Figure 1:**
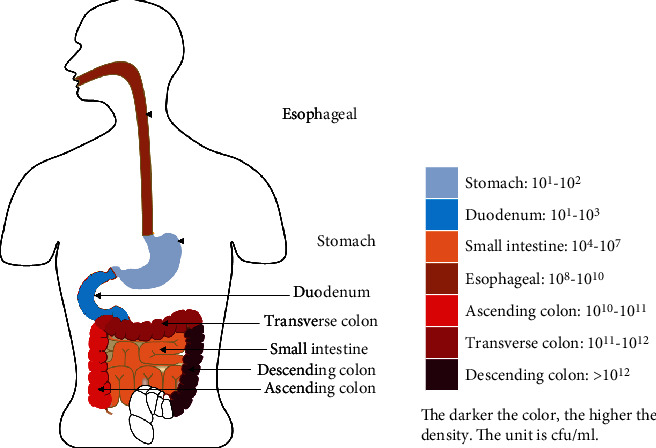
Bacteria count in different regions of the human digestive tract. The darker the color, the higher the density of bacteria. The unit is cfu/ml.

The gastrointestinal tract contains the largest microbiome in the human body, accounting for 80% of the total microbial biomass. For a long time, people believed that the stomach was sterile. By the early 1980s, Marshall and Warren isolated *Helicobacter pylori* (*H. pylori*) from gastric biopsies obtained from patients with chronic gastritis and peptic ulceration, for which they won the 2005 Nobel Prize in Physiology or Medicine for their discovery, after that the researchers were interested in the bacterial cause for ulcer disease [1]. With the advent of 16sRNA gene sequencing, next-generation sequencing technology, metagenomics, and other research methods, it is possible to identify gastric microbes and to explore the functional activities and interrelationship of gastric microbial communities. It has been found that in addition to *H. pylori*, there are other microorganisms in the stomach, which jointly form the microbial environment in the stomach and may be related to the occurrence of many gastrointestinal diseases. The total number of bacteria carried in the intestines of a healthy person is estimated to be 10^14^, which is constituting a microbiome and an ecosystem in dynamic balance as a whole [2]. In general, the number of bacteria from the stomach to the large intestine is different, and the bacterial concentration presents an increasing state successively. The count of bacteria in oral cavities is higher. There is the least count of bacteria in the stomach. The small intestine has more bacteria, while the colon has the most bacteria, which is nearly 10^10^ times more than the count of bacteria in the stomach [3, 4] (shown in Figure 1).

At present, there are few studies on microbes living in the stomach and duodenum, mainly due to its special physiological processes such as gastric acid secretion, bile secretion, and gastrointestinal motility. However, little is known about the relationship between microorganisms and these physiological processes and how they affect health and disease throughout the digestive tract. In this article, we reviewed advances in human gastric microecology, the relationship between gastric microecology and immunity or gastric diseases, and the treatment of probiotics in gastric diseases, in order to explore new area for further study of gastric microorganisms and treatment of gastric diseases.

## 2. Anatomy and Physiological Mechanism of the Stomach

**Figure 2:**
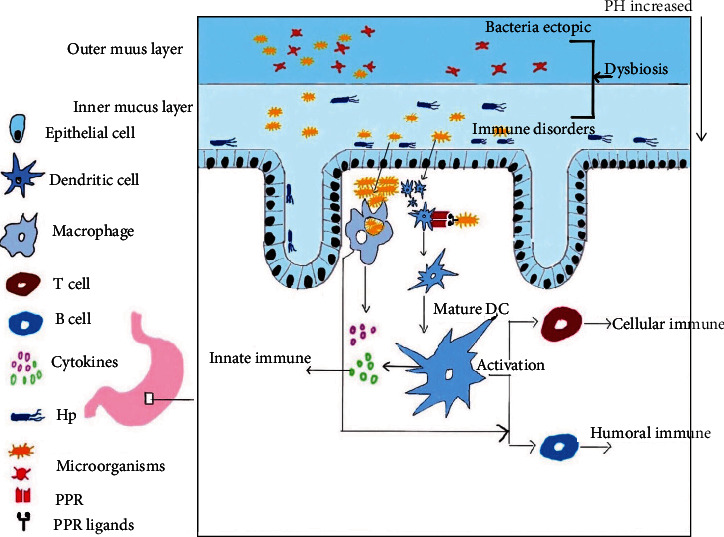
Schematic diagram of the cause for gastric mucosal immune disorder caused by microorganisms.

The stomach accommodates food, secretes gastric juice, digests food, and has a secretory function. Due to its special anatomical structure, the secretion of gastric acid and digestive enzymes, bile reflux, special mucus barrier, and gastric peristalsis all contribute to the formation of an antibacterial environment. This is also different from other digestive tracts. Normal gastric juice has a pH of 0.9-1.5 and its secretion is 1.5-2.5 L/d. During or after eating, the movement of the stomach is enhanced, and the secretion of gastric juice is increased. The stomach can also secrete other mucus, which covers the surface of the gastric mucosa, forming a protective gel-like layer. The mucous layer on the mucosal surface is divided into inner and outer layers, the pH of which gradually increases from the outer layer to the inner layer (shown in Figure 2).

When microecological imbalance in the stomach or other reasons cause damage to the first line of gastric mucosal immunity, which is composed of gastric mucosal epithelial cells and the mucous layer on the surface, the permeability between gastric epithelial cells increases, and pathogens and their metabolites enter the mucosa through the gap, which causes the macrophages to accumulate. Recognition of pattern recognition receptors (PPR) in dendritic cells and other immune cells can lead to direct macrophage phagocytosis and the production of cytokines by the cells mentioned above, which can stimulate T cells and B cells to produce cellular immunity and humoral immunity, and finally, further eliminate microorganisms through the innate immunity and adaptive immunity of the gastric mucosa. However, some microorganisms can achieve immune escape by targeting gene expression. Microecological imbalance can also lead to a decrease in dominant bacteria and an increase in the abundance and virulence of pathogenic microorganisms, thereby leading to the occurrence of diseases. Normal digestive motility is also essential to maintain the balance of microorganisms in the digestive tract. Impaired gastrointestinal motility can hinder the absorption of drugs and nutrients, resulting in immune function and intestinal mucosal integrity impairing, bacterial overgrowth and translocation, and the release of endotoxins into the circulation and activation of immune responses. On the other hand, bacterial overgrowth may also cause gastrointestinal dysfunction, such as gastroparesis and other gastro motility disorders. Due to the special structure and function of the stomach mentioned above, the growth of microorganisms in the stomach is restricted to physiological levels. If the pH, the mucosal barrier, or gastric physiological movement of the stomach are altered, the microecological balance in the stomach may be disturbed, which will affect its normal function and promote the occurrence of gastric diseases [5]. For example, histamine 2 receptor antagonist therapy or atrophic gastritis increases the growth of nitrosating bacteria, which can convert nitrite and other nitrogen compounds in gastric juice to carcinogenic N-nitroso compounds [6]. In healthy conditions, gut flora is involved in maintaining the integrity and function of the epithelial mucosal barrier, and gastrointestinal immune cells strengthen the epithelial barrier function by maintaining a healthy microbial community. Once the gastrointestinal immunity is unbalanced, it may lead to gastrointestinal dysfunction and diseases, including inflammatory bowel disease [7], irritable bowel syndrome [8], small intestinal bacterial overgrowth [9], B cell lymphoproliferative diseases [10], and allergic intolerances to foods [11]. Proper gastrointestinal motility allows for a constant flow of luminal materials through the gastrointestinal tract, which prevents bacterial overgrowth in the small intestine. However, patients with gastrointestinal dysmotility have a stagnant flow of luminal materials, leading to the development of small intestinal bacterial overgrowth (SIBO) [12].

## 3. Relationship between Gastric Microbe and Gastric Immune Regulation

The human immune system contains PPRs that distinguish harmful pathogenic microorganisms from harmless symbiotic ones. Toll-like receptors (TLRs) are kind of important PRR expressed in the macrophage and dendritic cell membrane, and the other PRR group is the nodal receptors (NLRs). NLRs are associated with a family of innate cytoplasmic receptors that are involved in the detection of intracellular pathogens and endogenous byproducts of tissue injury [13]. Microecological imbalance can contribute to the invasion of pathogenic microorganisms and can damage the mucosal barrier and the immune system. This process results in increased mucosal permeability, inflammation of the digestive tract, and thus, activation of TLR and NLR signals. When the antigen comes into contact with the human body, immune cells in the blood bind to chemokines and induce immune cells to adhere to the cell adhesion factor of endothelial cells by integrin and then migrate through endothelial cells to the stomach [14]. Finally, the body exerts immune function through innate immunity and adaptive immunity [15] and maintains microbial balance through the immune homeostasis mechanism [16] (shown in Figure 2).

Immune-related gastric mucosal cells are composed of gastric mucosal epithelial cells, macrophages, and dendritic cells. Gastric mucosal epithelial cells are located in the gastric mucosal epithelium, which together with mucous layer on the surface constitute the first line of defense for gastric mucosal immunity. Macrophage microaggregates are widely distributed in the gastric mucosa [17]. Cytokines generated after macrophage activation stimulate the occurrence of immune response, play an immunomodulatory role, and promote the occurrence of adaptive immune response [18, 19]. Dendritic cells exist in the human gastric mucosa. Mature dendritic cells are activated as antigen-presenting cells (APCs), which activate effector T and B cells through the TLR signal and induce an adaptive immune response [14, 20–24].

Gastric mucosal adaptive immunity-related cells are composed of T cells, B cells, and other immune lymphocytes. In gastric mucosal cell immunity, CD4+ T cells and regulatory cells play an important role [25, 26]. The humoral immunity of the gastric mucosa mainly involves B cells and immunoglobulin. B cells exert humoral immune function with the joint participation of macrophages, helper T cells, and chemokines [27]. The microbiota can damage host DNA and activate signal transduction, resulting in chromosomal aberrations, microbial translocation, and the activation of myeloid cells that produce interleukin (IL-23) and thus promoting tumor growth [28]. Some studies have shown that a high abundance of *Fusobacterium nucleatum* in the gastrointestinal tract can increase plasma proinflammatory cytokine levels and reduce the activity of NK cells [29]. In addition, *Fusobacterium nucleatum* can suppress accumulation of tumor infiltrating T cells and promote tumor growth and metastatic progression [30].

## 4. Microecological Environment of the Stomach

The fetus is aseptic when it is in the mother's body. It is in contact with the outside environment after birth, and many bacteria enter the body within a few hours. The presence of bacteria in the stomach may be due to the increasing pH from the gastric cavity (pH 1-2) to the mucosal surface (pH 6-7), while the mucosal surface is covered with mucus secreted by the gastric glands (shown in Figure 2). This pH gradient leads to a different environment in the stomach and allows the growth of microorganisms [31]. Since the proximal end of the stomach is connected to the esophagus and mouth and the distal end is connected to the duodenum, microbes from other parts of the human gastrointestinal tract can also enter the stomach.

*H. pylori* can cause a variety of stomach diseases. As an important pathogenic factor for chronic gastritis and gastric ulcer, *H. pylori* has been widely recognized and is closely related to the occurrence, development, and outcome of gastric cancer. Researchers have performed the first modern high-throughput sequencing study on stomach bacteria [32]. They characterized the gastric microbiota using PCR and 16S rDNA sequence analysis. They found that the human gastric environment contained rDNA from a wealth of bacteria, in addition to *H. pylori*. Of these, some were derived from uncultivated bacteria, and some had been previously described in specimens from the human mouth. Because it was likely that the composition of the gastric community was not only determined by niche-specific factors but also by stochastic colonization from upstream components of the alimentary tract.

Early studies have found microorganisms related to the gastric mucosa, such as *Enterococcus*, *Pseudomonas*, *Staphylococcus*, and *Stomatococcus* [33]. The composition of the gastric microbiota is highly variable between individuals. However, recent studies have identified five major phyla in the stomach, including *Firmicutes*, *Bacteroidetes*, *Actinomycetes*, *Clostridium*, and *Proteobacteria*. The main genera in the stomach include *Prevotella*, *Streptococcus*, *Roseburia*, and *Haemophilus* [34–39]. A systematic review of the gastric microbiota recently published also showed that the results of the gastric microbiota composition were highly heterogeneous. A total of 266 bacterial genera were identified, of which 57 were mainly found in normal acidic stomach [40].

Fungal flora can also be detected in the gastrointestinal tract, most of which are aerobes or facultative anaerobes, and the number of fungi in the human stomach ranges from 0 to 10^2^ cfu/ml [41]. *Candida albicans* can grow well in a highly acidic environment [42], and some genotypes can aggravate the severity of gastric mucosal lesions [43]. A study showed that 66.7% of patients with gastric diseases had colonization of *Candida* and *H. pylori* in the gastric mucosa [44]. Although a causal relationship with secondary colonization was never examined in these studies, the coexistence of *Candida albicans* and *H. pylori* may indicate a synergistic role in the pathogenesis of gastric ulcer, and the mycelium formed by *Candida* may contribute to ulcer perforation. Although fungi play an important role in the study of gastric microbiology, their potential role in the pathogenesis of diseases needs to be further studied with more modern techniques. At the same time, the composition of the gastric microbiota is affected by factors such as *H. pylori*, health status, diet habits, drug use, age, surgical intervention, and inflammation [45, 46].

## 5. Interactions between Microbes in the Stomach

The gastrointestinal microecosystem is a unity formed by the interaction and influence of the gastrointestinal flora, its host, and its external environment. The gastrointestinal flora maintains the stability and balance of the gastrointestinal microecosystem through a variety of regulatory systems and pathways. Once the balance is destroyed, there will be a microecological imbalance, which will lead to the generation of diseases. At present, many studies have found that non-*H. pylori* gastric microorganisms are related to gastric diseases, and *H. pylori*, which has been much more studied, also interacts with other gastric microorganisms.

The colonization of *H. pylori* in the gastric mucosa changes the gastric environment by decomposing the mucosal layer and alkalizing gastric juice [47]. Some studies have confirmed that non-*H. pylori* gastric microorganisms are related to the development of gastric cancer. In this study, transgenic Ins-GAS mice with gastrin overexpression were used. Ins-GAS mice treated with antibiotics developed gastric cancer later than control mice without antibiotic treatment for *H. pylori* [48], suggesting that gastric microorganisms may enhance the role of *H. pylori* in gastric cancer. Changes in gastric microbial ecology after *H. pylori* eradication indicate that *H. pylori* affects the interaction of other microorganisms in the stomach, possibly promoting the development of inflammation and cancer in patients [49]. Eradication of *H. pylori* can prevent the progression of gastric mucosal lesions [50, 51], but some patients continue to progress to precancerous lesions, including gastric atrophy (GA) and intestinal metaplasia (IM), after radical treatment with *H. pylori* [36]. Less than 3% of patients infected with *H. pylori* develop gastric cancer [52], and about 20% of patients with chronic gastritis are negative for *H. pylori*, suggesting that other microorganisms may induce gastritis and even gastric cancer [53]. Although *H. pylori* initiates the gastric inflammation process, other gastric microorganisms with proinflammatory potential may play an important role in maintaining the progression of inflammation and abnormal hyperplasia, which then leads to the development of gastric cancer.

The effect of *Lactobacillus salivarius* supplementation has been demonstrated in mouse models, and its effectiveness is related to the large amount of lactic acid produced by the bacteria that interferes with the urease activity of the pathogen [54]. Furthermore, *in vitro*, *L. salivarius* can reduce gastric inflammation by regulating local cytokine secretion, especially IL-8, which is directly related to neutrophil recruitment and mucosal inflammation, possibly in response to inhibiting the secretion of cytotoxin-associated protein virulence factor (Cag A) [55]. Multiple studies have found the presence of Epstein-Barr virus (EBV) and *H. pylori* in gastric cancer specimens (ranging from 6% to 12%). Meta-analysis has also evaluated the importance of co-infection of these two pathogens in gastric cancer [56–59]. Currently, different studies [56, 57, 60] have explored the possible roles of combined infection by *H. pylori* and EBV in the development of gastritis, peptic ulcer, dyspepsia, and gastro esophageal reflux disease (GERD). In the coinfection process of *H. pylori* and EBV, the recruitment of immune cells at the infected site significantly increase, thus aggravating gastric inflammation and tissue damage [61]. For example, monochloramine is an oxidant produced in the stomach during *H. pylori infection*, which can induce the transition of EBV from the latent phase to the cleavage phase [62], and *H. pylori* induces the secretion of interferon *γ* (IFN-*γ*), which promotes the inflammatory environment and exacerbates the severity of the disease [61]. The intestinal microbiota can regulate *H. pylori* infection, and vice versa, this bacterium can alter the composition of the stomach microbiota [63–65]. On the other hand, the microbiota in several human niches have a direct or indirect influence on viral infection, such as EBV and human papillomavirus [66].

Daily changes in intestinal flora composition have been observed after the administration of a drug used to eliminate *H. pylori* to rats, and it has been found that the number of obligate anaerobes is significantly reduced, the number of short-chain fatty acids (SCFAs) is reduced, and the stool is slightly abnormal [67]. *Clostridium difficile* has been reported to grow particularly well when strong and/or broad-spectrum antibiotics are used [68]. Administration of CBM588 probiotics at the same time as eradication of *H. pylori* can inhibit the production of *Clostridium difficile toxin A*, thus reducing the risk of diarrhea and soft stool in these patients [67]. The stomach microecology of healthy people is balanced, and a stable microenvironment is formed between microorganisms due to long-term symbiosis. The change in any kind of microorganism may have an impact on other microorganisms and microecology.

## 6. The Microecology of the Stomach Is Involved in the Occurrence of Gastric Diseases

*H. pylori* can hydrolyze urea to produce ammonia through urease, which in turn increases the local pH value and is conducive to the colonization of other microorganisms. Furthermore, both epigenetic and direct inheritance of *H. pylori* can directly lead to gene instability, including double-stranded DNA breakage [69, 70].

Gastric microorganisms can aggravate the histological changes caused by *H. pylori* infection. After *H. pylori* eradication, the atrophy score of 20.8% for subjects decreased [50]. Coinfection of *H. pylori* and *Neisseria subflava* has been reported to be associated with the formation of lymphatic follicles in the human stomach [71]. *Neisseria subflava* can induce IL-8 production by gastric epithelial cells and promote the progression of hypoacid-induced gastric function lesions. The occurrence of GA and IM leads to a reduction in gastric acid-secreting wall cells [72], which may be conducive to the proliferation of gastric microorganisms or the colonization of oral microorganisms in the stomach [73]. In the absence of *H. pylori* infection, the presence of atrophy or IM was associated with an increased abundance of *Granulicatella*, *Actinomyces*, *Rothia*, *Peptostreptococcu*s, *Streptococcus*, *Abiotrophia*, and *Parvimonas* [49]. A previously published study by Parsons et al. focused on evaluating the diversity of stomach microbes in various hydrochloric acid states, including *H. pylori*-induced atrophic gastritis and autoimmune atrophic gastritis (AIG). This study showed that the microbial diversity and bacterial abundance of AIG patients were higher than those of normal stomachs, and *Streptococcus* accounted for the largest proportion in the investigated group [74]. Currently, the study on the gastric microbiota of patients with AIG is still in an early stage. In subjects with gastric mucosal atrophy after removal of *H. pylori*, the number of *Faecalibacterium*, *Kaistobacter*, and *Rahnella* decreased [49]. Sjöstedt et al. studied microbial colonization in the oropharynx, esophagus, and stomach of 60 patients and found that patients with a history of gastritis, gastric cancer, and gastrectomy had more gastric microbes than patients with gastric or duodenal ulcers. Gastric cancer patients have the largest number of different microbial colonization [75].

Currently, there are many studies on the effects of viruses on the intestinal nervous system, and it is found that the effects of viruses on the intestinal nervous system can lead to gastrointestinal motor disorders. EBV infection is closely related to gastritis and gastric cancer [20, 57]. EBV can inhibit the proliferation of T cells and the toxicity of natural killer cells and maintain the activity of the virus in host cells, resulting in sustained damage to the gastric mucosa [76]. EBV can keep the virus at a very low expression level by targeting the gene expression of the virus and avoid the attack of the human immune response [76, 77]. Recently, some authors reviewed the changes of some microorganisms in gastric diseases [78] (shown in Table 1).

## 7. Microorganism and the Treatment of Gastric Disease

The modern history of probiotics begins at the beginning of the1900s, Nobel Prize winner Elie Metchnikoff found that when yoghurt or fermented milk containing *Lactobacillus bulgaricus* was consumed, the gastrointestinal condition improved significantly, and hence, the practice of using probiotics has arisen [89, 90].

According to the Food and Agriculture Organization/World Health Organization [91, 92], probiotics are living microbial agents that, when given in sufficient quantities, have a beneficial effect on the host. Many strains of lactic acid bacteria benefit the host by inhibiting the growth of pathogens and inhibiting inflammation, tumor, and allergic modification [93–96]. The beneficial effects of probiotic bacteria on host microbial environment may be due to their potential impact on the digestive tract microbial community and the intestinal immune system, including their ability to compete with intestinal pathogens, increase the secretion of Immunoglobin A (IgA), regulate the expression and secretion of cytokine, stimulate mucin, bacteriocins, and lactic acid production, and adjust the microbiota growth [97–99].

Some probiotics have not only preventive effects but also therapeutic effects by promoting epithelial cell growth [100] and angiogenesis [101] and upregulating the expression of anti-inflammatory cytokines [102]. Therefore, the addition of probiotics to patients undergoing radical treatment for *H. pylori* can prevent gastritis caused by other microorganisms [49]. Probiotics have a potential role in alleviating gastritis after radical treatment for *H. pylori* [103, 104]. *Lactobacillus* given to rats as a single probiotic strain, such as *Lactobacillus gasseri OLL2716* [105, 106], *Lactobacillus acidophilus* [100, 107], *Lactobacillus rhamnosus GG* [108], or in the form of probiotic mixtures, has been reported to promote ulcer healing. *Lactobacillus rhamnosus GG* improves the ratio of proliferation to apoptosis of host cells and causes continuous regeneration of epithelial cells, especially around the edge of ulcers [108, 109]. *Saccharomyces boulardii* has a good therapeutic effect on ibuprofen-induced gastric ulcer in rats [110]. The neuraminidase activity of the *Saccharomyces boulardii* can remove surface *α* (2-3)-linked sialic acid from apical cells of the gastric epithelium. Thus, by eliminating sialic acid, *H. pylori* mucin-mediated adhesion to gastric epithelial cells is prevented [111].

Acetic acid, propionic acid, and butyric acid are all SCFAs that are important energy sources in the body and are produced primarily in the colon as a result of the metabolization of indigestible carbohydrates by microorganisms [112], which can regulate energy balance through the brain-gut axis. Excess alcohol consumption damages the gastric mucosal barrier, leading to extensive hemorrhagic damage, accumulating oxidative stress, and increasing the inflammatory response through the production of cytokines such as interleukin-1*β* (IL-1*β*), interleukin-6 (IL-6), and tumor necrosis factor-*α* (TNF-*α*) [113]. Butyrate pretreatment can negatively regulate the proinflammatory cytokines IL-1*β*, TNF-*α*, and IL-6, enhance the function of gastric wall mucus [114], and has a protective effect on ethanol-induced gastric ulcer formation. *Lactobacilli* and *Bifidobacteria*, as the main genera of probiotics, have been shown to produce acetic acid *in vitro*, but cannot produce propionic acid and butyric acid as the main metabolites. However, these common probiotics can stimulate the production of SCFAs by other colonic bacteria, producing pyruvate and lactic acid from dietary carbohydrates [115]. Nagaoka et al. reported that *Bifidobacterium bifidum YIT 4007* improved acute gastric injury induced by ethanol and acetic acid in rats [116]. In addition, probiotics, prebiotics, and/or synbiotics can regulate the gut microbiome, thereby inhibiting pathogens and promoting the growth of SCFA-producing bacteria [117].

## 8. Conclusions

In recent years, with changes in diet and increased work pressure, the incidence of *H. pylori* infection and gastric ulcer is very high, and *H. pylori infection* is considered a primary risk factor for gastric cancer, and gastric ulcer also has a certain rate of malignant change, so it is particularly important to treat *H. pylori* infection and gastric ulcer. Most of the previous studies focused on *H. pylori* and relatively ignored other microorganisms in the stomach and the overall microecology of the stomach. At present, research on the correlation between gastric microorganisms is mostly limited to the relationship between *H. pylori* and non-*H. pylori* microorganisms. There is a lack of in-depth understanding for the improving effect of probiotics on gastric ulcers. We should consider microecology as a whole when we study microbes in the stomach in the future. In the treatment of microbial infection, blind sterilization is not recommended, leading to ignore the overall relationship between the stomach microbes. We should also consider the variety of factors that may affect the stomach microbiota, such as drugs, diet, smoking, and drinking habits. The ecological characteristics of different areas in the stomach are different, so the biome may be different. The stomach is not a single ecological environment, and different anatomical parts may have different microbial colonization. In general, these need to be further studied. At present, there is little direct evidence on the non-*H. pylori* microbiome in gastric ulcers, precancerous lesions, and gastric cancer. Future gastric microbiome research will include transcriptomics, metabolomics, and proteomics, which will provide more opportunities for functional studies of gastric microorganisms.

## Figures and Tables

**Table 1:** Changes in Microorganisms in Different Gastric Diseases.

Gastro-Related Diseases	Control Group	Changes in Gastric Microorganisms	Reference
Atrophic Gastritis	Healthy Subjects	Streptococcus↓, Prevotella↓	Engstrand L, et al. [79]
Gastric Cancer	Normal Mucosa	Microorganisms↑, Anaerobic Bacteria (eg Clostridium and Bacteroides species)↑	Dicksved J, et al. [80]
Chronic Gastritis	Normal Control Group	Prevotella↑, Streptococcus↑, Neisseria↑, Porphyromonas↑, Haemophilus↑	Nardone G, et al. [81]
H. Pylori-Infected Gastritis	H. Pylori-Negative Individuals	Proteobacteria↓, Firmicutes↑	Li XX, et al. [82]
Atrophic Gastritis	Healthy Controls	Streptococcus↑, Prevotella↓	Ozbey G, et al. [83]
H. Pylori-Infected Antral Gastritis	Without H. Pylori Infection	Proteobacteria↓, Prevotella↓, Firmicutes↑, Streptococcus↑	Liu J, et al. [84]
H. Pylori-Infected Peptic Ulcer	/	Streptococcus↑, Neisseria↑, Rothia↑, Staphylococcus↑	Bilello J, et al. [78]
Invasive Gastric Cancer	Without Cancer	Porphyromonas↓, Neisseria↓, Streptococcus Sinensis↓, Lactobacillus Coleohomonis ↑, Lachnospiraceae↑, Pseudomonas↑	Zhang S, et al. [85]
Gastric Cancer	/	Nitrate-reducing bacterial species reducing nitrate (including Neisseria, Clostridium, Staphylococcus, and Clostridium Colicanis)↑	Hsieh YY, et al. [86]
Gastric Cardia Adenocarcinoma	/	Firmicutes, Bacteroidetes and Proteobacteria at the phylum level	Shao D, et al. [87]
Gastric Cancer	Chronic Gastritis	Achromobacter↑, Citrobacter↑, Phyllobacterium↑, Clostridium↑, Rhodococcus↑, Lactobacillus↑	Ferreira RM, et al. [88]
Gastric Cancer	Non-Tumor Tissues	Prevotella↑, Streptococcus↑, Veillonella↑, Haemophilus↑, Neisseria↑	Shao D, et al. [87]
